# Role of GnRH Neurons and Their Neuronal Afferents as Key Integrators between Food Intake Regulatory Signals and the Control of Reproduction

**DOI:** 10.1155/2013/518046

**Published:** 2013-09-11

**Authors:** Juan Roa

**Affiliations:** ^1^Department of Cell Biology, Physiology and Immunology, University of Córdoba, Avenida Menéndez Pidal s/n, 14004 Córdoba, Spain; ^2^CIBER Fisiopatología de la Obesidad y Nutrición, Instituto de Salud Carlos III, Spain; ^3^Instituto Maimónides de Investigaciones Biomédicas (IMIBIC)/Hospital Universitario Reina Sofia, Córdoba, Spain

## Abstract

Reproductive function is regulated by a plethora of signals that integrate physiological and environmental information. Among others, metabolic factors are key components of this circuit since they inform about the propitious timing for reproduction depending on energy availability. This information is processed mainly at the hypothalamus that, in turn, modulates gonadotropin release from the pituitary and, thereby, gonadal activity. Metabolic hormones, such as leptin, insulin, and ghrelin, act as indicators of the energy status and convey this information to the reproductive axis regulating its activity. In this review, we will analyse the central mechanisms involved in the integration of this metabolic information and their contribution to the control of the reproductive function. Particular attention will be paid to summarize the participation of GnRH, Kiss1, NPY, and POMC neurons in this process and their possible interactions to contribute to the metabolic control of reproduction.

## 1. Introduction

From an evolutionary point of view, the integration of the circuits controlling metabolism and reproduction is essential for the survival and perpetuation of the species. Wasting of energy on reproduction, in situations of deficit in food availability, may threaten the survival of the individuals and their progeny. Reproduction is a costly process in terms of energy consumption. Thus, in times of famine or under certain pathological conditions, which cannot ensure the correct utilization of the metabolic resources (anorexia nervosa, obesity, diabetes, lipodystrophies, etc.), the body has to define priorities to preserve physiological processes essential for survival [1]. In this sense, key physiological activities as blood circulation or neural activity cannot be compromised, whereas others, such as locomotion, thermoregulation, or growth can be reduced in conditions of metabolic stress, as occurs in hibernating animals.

In the above situations, reproduction is totally dispensable or even incompatible with survival since, besides the high energetic cost required to maintain the fertility, pregnancy and nursing limit considerably the mobility for food seeking. In wildlife, animals are more dependent on seasonal fluctuations in food availability than their counterparts living under domestic conditions or humans. However, the increased prevalence in developed countries of metabolic pathologies (ranging from anorexia to obesity and metabolic syndrome) makes the study of the metabolic control of reproduction of special interest, in part due to economic reasons. In this sense, it is interesting that the increase in the incidence of metabolic disorders during the last decades coincides also with increased rates of infertility, although direct association between these two phenomena has not been demonstrated so far [2, 3]. Of note, besides the elevated cost derived from the treatment of illness associated with metabolic disorders such as diabetes, hypertension, and other cardiovascular problems, the cost of a successful treatment by means of *in vitro* fertilization ranges from 19,588 to 134,190*€* [4].

Changes in energy stores produce long- and short-term fluctuations in hormonal (leptin, insulin, and ghrelin) as well as nutritional (glucose, lipids) signals that feedback mainly to the CNS to regulate metabolism and fertility. However, although many of the neural circuits controlling energy homeostasis are well characterized, the elements conveying nutritional information to the reproductive axis are yet to be conclusively defined. 

The reproductive axis comprises three major elements, the hypothalamus, the pituitary, and the gonads, which form the so-called HPG axis. Gonadotropin-releasing hormone (GnRH) neurons, located in the preoptic area (POA) of the hypothalamus, induce gonadotropin stimulation at the pituitary, which subsequently increases gonadal hormone secretion. The coordinated actions of these elements, together with other peripheral factors, allow the integration of endogenous and environmental information to ultimately produce gametes and modulate sex behaviour. 

## 2. GnRH Neurons and the Metabolic Control of Reproduction

GnRH neurons are located in an excellent position to be considered as the best candidate to relay metabolic information to the downstream elements of the HPG axis since (i) GnRH neurons are the final output of the brain controlling the reproduction, (ii) GnRH neurons can sense peripheral signals because they send multiple projections to the *organum vasculosum of the lamina terminalis* (OVLT) and median eminence where the permeability of the blood-brain barrier is very high, and (iii) GnRH mRNA and the patterns of GnRH secretion change in situations of metabolic stress, suggesting that GnRH neurons are influenced by nutritional reserves.

However, there is a growing body of evidence demonstrating that GnRH neurons may not be targeted directly by the above metabolic factors. In support of this idea, recent analyses demonstrated that the signal transducer and activator of transcription 3 (STAT3) is not presented in GnRH neurons after leptin administration, and that GnRH neurons lack leptin receptor (LepR), assessed by single-cell PCR. Further proof for the absence of direct actions of leptin on GnRH neurons came from functional genomic studies involving selective ablation of LepR in GnRH neurons or in all forebrain neurons. These experiments demonstrated that elimination of leptin signalling in GnRH neurons does not have any impact on reproduction, whereas LepR ablation in all forebrain neurons impaired fertility [5]. Along with leptin, insulin is another potential candidate to relay metabolic information to the reproductive system. In fact, neuron-specific insulin receptor-knockout (NIRKO) mice exhibit hypogonadism of central origin [6]. However, the direct influence of insulin on GnRH neurons or even the presence of insulin receptors (IR) in this neuronal population has been extensively questioned. Recent analyses have confirmed that, although IR is expressed in GnRH neurons, the selective ablation of IR on this neuronal population, by breeding IR^fl/fl^ mice onto a GnRH-Cre background, does not alter the normal timing of puberty or fertility [7]. These data suggest that the positive effect of insulin on reproduction is not mediated directly via GnRH neurons. 

Contrary to leptin and insulin, ghrelin is a signal of energy deficiency. Compelling evidences have highlighted the negative effect of ghrelin at different levels of the reproductive axis [8]. In this sense, chronic administration of ghrelin to peripuberal rats inhibits gonadotropin release and mimics the delay on puberty onset produced by situations of energy deficit, such as chronic undernutrition [9], conditions in which endogenous ghrelin levels are expected to be elevated. Besides its central effects, it has been demonstrated that ghrelin can also modulate the reproductive function through its actions on the pituitary and gonads [8, 10]. Ghrelin receptor GHS-R1a mRNA is present in many areas of the brain. However, conclusive demonstration of the expression of this receptor in GnRH neurons remains elusive. Nonetheless, studies in rats of the inhibitory effects of ghrelin on GnRH pulsatility (as evidenced by an increase of GnRH interpulse intervals) demonstrated that this can be blockaded by an NPY Y5R antagonist [11]. This suggests that ghrelin effects on GnRH neuron are mediated, at least in part, by afferent neurons (probably NPY neurons). 

The above lines of evidence indicate that, although GnRH neurons are ultimately affected by metabolic signals, some key peripheral indicators of the energy status do not target this information directly on GnRH neurons. Some of the potential candidate afferents responsible for transmitting metabolic information to GnRH neurons will be reviewed in the following sections. 

## 3. Kiss1 Neurons and the Metabolic Control of Reproduction

Kisspeptins, encoded by the *Kiss1* gene, are peptides derived from a common precursor named kisspeptin-54, which was originally termed metastin by its ability to suppress melanoma metastasis [12]. These peptides share a common RF-amide carboxyl-terminal region, which is essential for the activation of its G-protein-coupled receptor, GPR54. Both *Kiss1* and *GPR54* genes have been phylogenetically well conserved, certifying the importance of this system [13]. In 2003, two independent studies uncovered the indispensable role of kisspeptins and its receptor for the normal timing of the maturational events controlling the reproductive function. These two reports identified that inactivating mutations of GPR54 causes hypogonadotropic hypogonadism, suggesting that Kisspeptin signaling plays a major role in the control of the reproductive axis [14, 15]. Since then, a large number of papers have confirmed and extended this initial observation, thus deepening into the molecular and physiological mechanisms involved in the control of the reproductive function by the Kiss1/GPR54 system. 

In rodents, Kiss1 neurons are organized in two different populations neuroanatomically separated within the hypothalamus. One of these populations is located in the so-called rostral periventricular area of the third ventricle (RP3V), which comprises the anteroventral periventricular nucleus (AVPV), the rostral preoptic periventricular nucleus (rPVpo), and the caudal preoptic periventricular nucleus (cPVpo) [16]. Several studies have demonstrated that this population is activated by estradiol and sends projections to GnRH neurons in the POA suggesting that RP3V Kiss1 neurons are potentially involved in conveying the estrogen-positive feedback [17, 18]. The other population, located in the arcuate nucleus (Arc), shows diametrically opposite responses to estradiol, as Arc *Kiss1* expression is inhibited by estrogen. This circumstance, together with the fact that Arc Kiss1 neurons also project (albeit to a lesser extent) to GnRH neurons in the POA, suggests that this neuronal population could be involved in mediating estrogen-negative feedback [19, 20]. Of note, however, although this assumption has been accepted for years, recent data from electrophysiological recordings have failed to document electrical activation of Arc Kiss1 neurons after withdrawal of the inhibitory effect of gonadal steroids in mice [21]. 

Kiss1 neurons have been also proposed as mediators for relaying metabolic information to GnRH neurons [22]. There are compelling evidences supporting this hypothesis. Fasting, which reduces significantly LH secretion, is correlated with diminished *Kiss1* expression [23–25]. Chronic administration of kisspeptin partially rescues the delay on puberty onset caused by chronic undernutrition [26]. Anovulation and reduced levels of GnRH/LH during lactation, caused by the negative energy balance due to milk production, are associated with reduced levels of *Kiss1* mRNA in the Arc [27]. Infertile leptin-deficient mice (ob/ob) show low levels of *Kiss1* mRNA, and leptin injection recovers, in some extent, *Kiss1* expression and fertility [28]. Likewise, streptozotocin-induced diabetic rats, which are leaner and hypoleptinemic, display low levels of *Kiss1* mRNA in the hypothalamus. However, chronic injections of Kisspeptin-10 partially rescued the negative effects of insulin deficiency on reproductive parameters (LH and testosterone secretion and prostate and testis weights) [29]. All in all, the above data point out that conditions of negative energy balance and metabolic stress that suppress the function of the HPG axis are associated with a detectable inhibition of the hypothalamic Kiss1 system.

Despite this solid evidence, whether Kiss1 neurons are direct targets for metabolic factors is still under discussion. Although previous studies assumed that almost half of the arcuate Kiss1 neurons express LepR [28], recent data demonstrated that the ablation of this receptor on Kiss1 neurons or its restoration on LepR-deficient mice does not have a detectable impact on reproductive function [30, 31]. In fact, a recent paper suggested that only a low percentage of Kiss1 neurons expresses LepR, only after completion of puberty onset. These results suggest that leptin may not act directly on Kiss1 neurons to control the reproductive function. However, it is highly plausible that leptin could modulate the activity of Kiss1 neurons through afferent inputs. In this context, it has been demonstrated that leptin signalling in the ventral premmamillary nucleus (PMV) is essential for puberty and fertility in mice, and LepR-expressing cells in this nucleus send projections to Kiss1 neurons [30]. This arises the possibility that Kiss1 neurons could act as downstream mediators for leptin actions on the PMV [32]. In this sense, recent data demonstrated that targeted lesion of the PMV disrupts *Kiss1* and *GnRH* expression during the proestrus-to-estrus transition in rats [33].

On the other hand, recent analysis revealed that Kiss1 neurons express IR, suggesting that Kiss1 neurons could sense metabolic status by receiving information about insulin fluctuations in bloodstream, to regulate reproduction. In fact, IR ablation from Kiss1 neurons, by Cre-loxP strategy, caused a moderate delay of puberty onset and a reduction of LH secretion in both sexes. However, lack of IR on Kiss1 neurons did not affect fertility in adults [34]. These data suggest that insulin signalling in Kiss1 neurons exerts, to some extent, a positive influence for the initiation of the puberty, although in absence of this stimulus, compensatory mechanisms are activated to complete the maturation of the reproductive axis. 

In addition, the orexigenic hormone, ghrelin, which may play a major role disrupting fertility in situations of energy insufficiency, has been demonstrated to modulate *Kiss1* expression [35, 36]. Of note, intravenous injection of ghrelin has been shown to produce a significant decrease in LH pulsatility and *Kiss1* expression in the median POA (an area that includes the RP3V) of adult rats. These data suggest that Kiss1 neurons could contribute to mediate the negative effects of ghrelin on reproduction. However, whether Kiss1 neurons are direct targets for ghrelin remains to be clarified.

The above lines of evidence situate Kiss1 neurons as an excellent candidate responsible for the integration of metabolism and reproduction. However, many of the responses to metabolic hormones such as leptin, insulin, and ghrelin could be mediated by afferent inputs to Kiss1 neurons rather than by direct actions on this neuronal population.

## 4. Proopiomelanocortin Neurons and the Metabolic Control of Reproduction

Proopiomelanocortin (POMC) is a precursor protein that can be cleaved at different sites to generate several peptides with different biological activity. Among others, *α*-MSH, *β*-Endorphin, and ACTH are neuropeptides derived from the POMC precursor. In the brain, POMC neurons are divided into two different populations. One of them is located in the nucleus of the solitary tract (NTS). This is a small population comprised by nearly 190 neurons scattered throughout the dorsomedial and medial parts of the NTS [37]. The function of this neuronal population is still not well known. A recent study suggested that this population could be involved in short-term feeding suppression. However, selective ablation of NTS POMC neurons does not reproduce the obese phenotype showed by the global POMC-null mice [38, 39]. In addition, it has been demonstrated that these neurons do not respond to leptin [37]. Such evidences suggest that POMC neurons located in the NTS may have a marginal contribution to the global control of the energy homeostasis exerted by the POMC system. Further analysis will be necessary to unveil the function of the NTS POMC neurons. 

The other population is located in the Arc. Unlike NTS POMC neurons, the function and phenotype of Arc POMC neurons have been extensively studied. This neuronal population coexpresses multiple neuropeptides (CART, dynorphin, and VGF) and neurotransmitters (GABA, glutamate, acetylcholine) [40–43]. In addition, Arc POMC neurons express a wide range of receptors, such as LepR, IR, and NPY Y1-R, that confer the ability to sense peripheral and central signals involved in the control of metabolic homeostasis. This feature makes these neurons essential for the maintenance of energy status. In fact, selective ablation of Arc POMC neurons mimics the phenotype of the global POMC-null mice, producing increased food intake and reduced energy expenditure, resulting ultimately in obesity [38]. These lines of evidences situate POMC neurons as a central node for sensing body energy reserves and thus as key elements to finely tune the mechanisms involved in the control of food intake and energy expenditure to keep energy homeostasis. Accordingly, POMC mRNA expression in the Arc is reduced in leptin-deficient ob/ob mice, and leptin administration rescues this expression to that found in control mice [44]. Also, LepR deletion from POMC-expressing neurons disrupts body weight homeostasis, synapsis plasticity and produces hyperleptinemia [45, 46]. In the same vein, although IR ablation in POMC neurons does not cause any impact on body weight or glucose regulation [47], probably due to compensatory mechanisms, double LepR and IR deletion on POMC neurons produce higher negative impact on metabolic parameters than that found in the LepR KO mice [48]. 

The above lines of evidence demonstrate that Arc POMC neurons are key elements in the control of body weight and metabolism. This observation makes also POMC neurons a good candidate to relay metabolic information to GnRH neurons. Admittedly, information about the role of POMC products in the control of the reproductive axis is scarce and, in some cases, controversial. Yet, there is growing evidence suggesting that POMC neurons do participate in conveying metabolic information to GnRH neurons. In this context, whereas early studies demonstrated that independent ablation of LepR or IR on POMC neurons does not disrupt fertility [45, 47], mutant mice lacking both receptors in POMC neurons show severe reproductive deficiencies [48]. Moreover, immunohistochemical analyses demonstrated that POMC neurons project and make synaptic contacts with GnRH perikarya and nerve terminals, suggesting direct actions of POMC-derived peptides on GnRH excitability [49, 50]. 

Overall, the above data suggest that arcuate POMC neurons could act as a mediator for leptin and insulin actions upon GnRH neurons. In this sense, Watanobe showed, using push-pull perfusion techniques, that leptin infusion in the POA and median eminence stimulates GnRH/LH release, and this effect is preceded by an increase in *α*-MSH secretion [51]. Early pharmacological studies revealed that *α*-MSH is able to elicit a robust increase in LH in different mammalian species [52–54], as well as to stimulate sexual receptivity and lordosis behaviour in female rats [55]. However, discrepancies about the effect of *α*-MSH on LH can be found in the literature. In this context, there are data showing diminished or unchanged LH levels after *α*-MSH administration in rats [56, 57]. From these studies, it is apparent that steroid environment and administration site could influence LH response to *α*-MSH [58]. In this sense, recent data demonstrated that melanocortin receptors 3 and 4 (MC3/4R) agonist, Melanotan II, increases GnRH pulse generator activity in goats and this effect can be attenuated by estradiol [59]. This finding also suggests that the central actions of *α*-MSH on reproduction are mediated probably via MC3R and MC4R. 

Unlike the clear role of MC3R and MC4R on metabolism, evidenced by the fact that MC4R KO and to a lesser extent MC3R KO mice develop obvious metabolic disorders [60, 61], its role in reproduction remains uncertain. Data derived from MC4R mutants demonstrated that although MC4R-deficient mice are fertile, females are poor breeders exhibiting reduced ovulation rates [62]. Moreover, males display erectile dysfunction and disturbed copulatory behaviour [63]. In the same way, MC3R KO males are fertile though females display a certain degree of subfertility [61]. Overall, it seems that although both MC3R and MC4R are involved in the control of reproduction, the lack of one type of receptor could be partially compensated by the other, resulting in a milder phenotype. Accordingly, mice with functional blockade of both MC3R and MC4R signalling pathways, by overexpression of its endogenous antagonist, Agouti-related peptide (AgRP), are infertile [64]. 

However, whether GnRH neurons are direct targets of melanocortin actions remained unknown until very recently, when electrophysiological recordings of GnRH neurons demonstrated that *α*-MSH increases cell firing in most of GnRH neurons through postsynaptic activation of both MC3R and MC4R [65]. Almost simultaneously, Israel et al. confirmed by single-cell RT-PCR that GnRH neurons express MC4R and showed that restoration of melanocortin signalling in leptin-deficient db/db mice recovers the normal timing of puberty onset and fertility, suggesting that melanocortin signalling is essential for leptin actions on GnRH neurons [66]. In good agreement, our preliminary studies have documented that the positive effects of leptin on puberty onset, in rats subjected to 20% daily caloric restriction, can be blocked to a large extent by the MC3/4R antagonist SHU9119 (manuscript in preparation). Overall, it seems that POMC neurons convey leptin actions on GnRH neurons directly through MC3/4R pathways. In any case, the existence also of indirect intermediaries between POMC and GnRH neurons cannot be discarded. 

Although *β*-Endorphin is also derived from the POMC precursor, lines of evidence in the literature attribute to these neuropeptide effects diametrically opposite, in terms of metabolic and reproductive control, to those found for other POMC-derived peptides (such as *α*-MSH, *γ*-MSH, and ACTH). *β*-Endorphin mediates its actions mostly via *μ*-opioid receptor, although it displays also relatively high affinity by *δ*-, and *κ*-subtypes of opioid receptors [67]. Pharmacological experiments indicated that *β*-Endorphin increases food intake and body weight gain, whereas the opioid receptor antagonist, naloxone, inhibits feeding behaviour [68, 69]. Albeit there is consensus in the pharmacological studies about the stimulatory actions of *β*-Endorphin on food intake, paradoxically, mutant male mice retaining all the POMC-derived peptides except *β*-Endorphin show increased food consumption and are obese [70].

So far, pharmacological studies have shown mainly inhibitory actions for *β*-Endorphin on reproduction. Several analyses revealed that *β*-Endorphin inhibits basal GnRH/gonadotropin secretion in different species and physiological conditions [71–75], as well as the electrical activity of a subpopulation of murine GnRH neurons [65]. Also, central injection of *β*-Endorphin is able to block the preovulatory surge of LH [76] and to inhibit sexual behaviour [77]. Accordingly, naloxone administration consistently increases LH release [78–80]. Interestingly, naloxone administration to amenorrhoeic women is able to elicit a potent LH response, suggesting that excessive opioid activity could be involved in some pathophysiological conditions resulting in amenorrhea [81]. Intriguingly, mutant mice lacking *β*-Endorphin display a normal reproductive phenotype, showing normal puberty onset and fertility [82], which could be attributed to compensatory mechanisms mediated by other opioids.

The fact that *β*-Endorphin is able to negatively modulate GnRH/gonadotropin release opens up the possibility that POMC neurons could integrate also negative inputs to suppress GnRH activity via this opioid. In this sense, recent data suggested that the signal of energy insufficiency, ghrelin, suppresses the reproductive axis via *β*-Endorphin. Thus, Ogata et al. showed recently that central administration of ghrelin reduces significantly LH concentration and pulse frequency, whereas naloxone is able to block this effect [83]. However, whether *β*-Endorphin acts directly on GnRH neurons or via other intermediate neurons is still under discussion. In this context, while electrophysiological recordings in guinea pig demonstrated that the *μ*-opioid receptor agonist, DAMGO, inhibits GnRH neurons postsynaptically [84], data from the teleost fish, medaka, indicated that *β*-Endorphin reduces action potential firing in GnRH neurons via indirect mechanisms [85]. On the other hand, there is a large number of data demonstrating that rat GnRH neurons do not express opioid receptors [86, 87]. Admittedly, part of the above discrepancies could be attributed to interspecies differences. Regarding the possible indirect effects of *β*-Endorphin on GnRH neurons, pharmacological analyses suggested that *β*-Endorphin modulates GnRH release via glutamate-nitric oxide pathway [88].

CART-immunoreactive neurons have been identified in many different hypothalamic (paraventricular, arcuate, dorsomedial, and ventral premmamillary nuclei, as well as lateral hypothalamic area) and extrahypothalamic nuclei (central amygdala) [89–91]. However, the Arc POMC/CART population has received special attention due to its potential role in regulating food intake and reproduction, while little is known about the intervention of other CART populations in the integration of these two systems. 

Central administration of CART dramatically suppresses food intake [92, 93]. However, the receptor responsible for CART actions has not been identified yet. Unlike other Arc POMC neuropeptides, evidence for CART actions on the reproductive axis remains rather scarce. Nonetheless, data from *in vitro* incubation of hypothalamic explants showed that both CART and leptin are able to stimulate GnRH secretion by reducing interpulse intervals. Interestingly, coadministration of an anti-CART antiserum completely blocked CART effects on GnRH pulsatility whereas partially abrogated leptin actions [94, 95]. This means that Arc POMC neurons can mediate leptin actions on GnRH neurons, at least partially, through CART secretion. Intriguingly, very recent analysis demonstrated that CART could stimulate GnRH excitability both postsynaptically and presynaptically. The latter, probably via indirect actions through Kiss1 neurons [96]. 

Altogether, the above lines of evidence suggest that POMC neurons are key elements for conveying a large variety of metabolic inputs, ranging from signals of nutrient deficiency to energy sufficiency cues, to control the reproductive axis by secreting a wide diversity of neuropeptides with different, in many cases opposite, actions.

## 5. NPY Neurons and the Metabolic Control of Reproduction

NPY is member of a family of peptides that include also peptide YY (PYY) and the pancreatic polypeptide (PP). In rats, NPY-expressing neurons are widespread through different areas across the brain including, among others, the olfactory bulb, striatum, hypothalamus, spinal cord, and pineal gland, being the Arc and the paraventricular nucleus, within the hypothalamus, two of the nuclei containing higher concentrations of NPY neurons and fibers [97]. The Arc population coexpresses also AgRP and has been postulated as key element in the control of feeding behaviour [98]. In fact, in 2011 Aponte and co-workers demonstrated in an extremely elegant study that activation of arcuate NPY/AgRP neurons, using optogenetic tools, produces rapid stimulation of seeking behaviour and compulsive feeding in mice [99]. Interestingly, although AgRP is the endogenous antagonist of MC3/4R, this effect was demonstrated to be independent of melanocortin signalling suppression. These evidences suggest that Arc NPY neurons are essential to stimulate food consumption in situations of negative energy balance. 

So far, five different NPY receptors (Y1, Y2, Y4, Y5, and Y6) have been identified, which show different affinity for the various peptides of the NPY family [100–103]. These receptors present different distribution patterns within the brain and participate in the regulation of multiple functions [104, 105].

Regarding the potential role of NPY in reproduction, Arc NPY neurons send projections to GnRH perikarya and nerve terminals and have been suggested to participate in conveying information about energy insufficiency to the reproductive axis [106]. In fact, food restriction markedly increases NPY mRNA, and this is well correlated with reduced LH release [107]. In addition, the infertile phenotype displayed by ob/ob mice has been associated with high levels of NPY mRNA. Indeed, deficiency of either NPY or its Y1 or Y4 receptors in these animals rescues fertility [105]. Despite these compelling lines of evidence, the regulation of the gonadotropic axis by NPY is rather complex. In fact, pharmacological studies showed opposite effects of NPY on LH release depending on the steroid milieu and receptor agonist used. Thus, whereas NPY inhibited LH release in intact and castrated animals [108, 109], this neuropeptide induced opposite stimulatory effects on steroid-primed ovariectomized rats [110]. Interestingly, NPY KO mice do not display any reproductive alterations under normal conditions. However, fasting does not induce the expected decay in LH levels in these animals, suggesting that NPY signalling is needed for transmitting metabolic information when the conditions for reproduction are unfavourable due to reduced energy availability [111].

A recent electrophysiological study evaluated the role of NPY receptors on GnRH neuronal activity by subtractive analysis. To address such a wide spectrum of potential effects, a selection of NPY receptor agonists was used to determine the possible influence of each individual receptor on GnRH activity. This procedure allowed to identify that Y1R activation inhibits murine GnRH neurons [65]. In good agreement, previous data showed that Y1R activation significantly decreases the number of calcium transients in GnRH neurons from nasal explants [112]. Interestingly, the same inhibitory effect was also described in rats through Y5 receptor [113]. So, differences between species may exist for the type of receptor that mediates the inhibitory actions of NPY on GnRH neurons. On the other hand, Y4 receptor activation by different agonists resulted in a potent postsynaptic stimulation of GnRH neurons. Overall, the former discrepancies about the dual inhibitory/stimulatory effect of NPY on LH secretion could be due to differences in the ratio Y1R/Y4R in the model evaluated. Further analysis will be necessary to clarify this phenomenon.

As it was mentioned before, Arc NPY neurons also co-express AgRP. Both neuropeptides show similar response to fluctuations in energy availability. In this sense, AgRP mRNA is also increased in fasting conditions in order to stimulate food intake [114]. However, the mechanisms through which NPY and AgRP influence feeding behaviour are different. Accordingly, AgRP increases food intake, at least partially, by blocking the anorexigenic effect of endogenous MC3/4R activation by melanocortins [115]. 

Taking in account the clear stimulatory effect exerted by melanocortins on reproduction, opposite results might be expected for AgRP. In fact, early analysis showed that AgRP suppresses LH pulsatility in ovariectomized monkeys [116]. In addition, AgRP ablation in ob/ob mice rescued fertility [117]. Moreover, electrical recordings in GnRH neurons demonstrated that AgRP administration prevents the excitatory effect of the MC3/4R agonist, Melanotan II [66]. However, later results revealed that AgRP exhibits also stimulatory effects in a small subpopulation of GnRH neurons [65]. This paradoxical effect could be mediated via a mechanism independent of MCR3/4 receptors, a possibility that had been already considered in the context of other studies, which showed AgRP actions unexplained via MCR3/4 blockade [118, 119].

Overall, the above data suggest that Arc NPY neurons are able to module reproductive function through different mechanism involving NPY secretion and/or inhibition of melanocortin signalling by AgRP in situations of energy deficiency. 

## 6. Conclusions

Reproductive function is highly dependent on nutrient availability. To ensure an efficient utilization of the energy stores, redundant pathways are necessary. These circuits must detect situations of metabolic stress as to be able to derive energy resources to maintain essential physiological functions, while partially or totally suppressing reproduction until more favourable conditions are achieved. Of note, data in the literature suggest that many of metabolic signals informing the reproductive brain do no act directly on GnRH neurons, the final output in the brain controlling reproduction. Thus, afferent inputs are necessary to transmit metabolic information to GnRH neurons. In fact, in recent years, the existence of different neuronal populations that are able to sense peripheral and central indicators of the energy status to convey this information to GnRH neurons has been exposed by a large number of experimental studies. As reviewed herein, Arc POMC, NPY, and Kiss1 neurons have been proposed as key intermediary elements to carry out this function. Interestingly, there are solid lines of evidence suggesting that these neurons make direct contacts and are able to modulate the activity of each other [96, 120, 121]. This raises the possibility of the existence of a complex network of interconnected neurons that is involved in the precise sensing of the metabolic status and in the transmission of this information to GnRH neurons to consequently modulate the reproductive function. On the basis of the evidence summarized here, it is tenable to postulate that Kiss1, NPY, and POMC neurons are prominent elements of such a complex neuronal network. Admittedly, however, although for sake of concision this review has focussed only in this selected group of neuronal populations, it is likely that other partners exist on such circuitry responsible for central metabolic-reproductive interactions, such as neurons expressing galanin-like peptide (GALP), melanin-concentrating hormone (MCH), orexins, or corticotropin-releasing hormone (CRF) [122, 123]. While the evidence so far available suggests that the roles of the latter neuropeptides in the control of the HPG axis are less prominent, it remains a challenge for the future to decipher how major and subordinate metabolic regulators interplay with and impinge on the central elements of the reproductive axis. 
